# Nadir oxygen delivery is associated with postoperative acute kidney injury in low-weight infants undergoing cardiopulmonary bypass

**DOI:** 10.3389/fcvm.2022.1020846

**Published:** 2022-12-16

**Authors:** Peng Gao, Yu Jin, Peiyao Zhang, Wenting Wang, Jinxiao Hu, Jinping Liu

**Affiliations:** Pediatric Cardiac Surgery Center, National Center for Cardiovascular Diseases, Fuwai Hospital, Chinese Academy of Medical Sciences and Peking Union Medical College, Beijing, China

**Keywords:** acute kidney injury, cardiopulmonary bypass, cardiac surgery, infants, oxygen delivery

## Abstract

**Background:**

Acute kidney injury (AKI) is common after cardiac surgery with cardiopulmonary bypass (CPB) and is associated with increased mortality and morbidity. Nadir indexed oxygen delivery (DO_2_i) lower than the critical threshold during CPB is a risk factor for postoperative AKI. The critical DO_2_i for preventing AKI in children has not been well studied. The study aimed to explore the association between nadir DO_2_i and postoperative AKI in infant cardiac surgery with CPB.

**Methods:**

From August 2021 to July 2022, 413 low-weight infants (≤10 kg) undergoing cardiac surgery with CPB were consecutively enrolled in this prospective observational study. Nadir DO_2_i was calculated during the hypothermia and rewarming phases of CPB, respectively. The association between nadir DO_2_i and postoperative AKI was investigated in mild hypothermia (32–34°C) and moderate hypothermia (26–32°C).

**Results:**

A total of 142 (38.3%) patients developed postoperative AKI. In patients undergoing mild hypothermia during CPB, nadir DO_2_i in hypothermia and rewarming phases was independently associated with postoperative AKI. The cutoff values of nadir DO_2_i during hypothermia and rewarming phases were 258 mL/min/m^2^ and 281 mL/min/m^2^, respectively. There was no significant association between nadir DO_2_i and postoperative AKI in patients undergoing moderate hypothermia during CPB.

**Conclusion:**

In low-weight infants undergoing mild hypothermia during CPB, the critical DO_2_i for preventing AKI was 258 mL/min/m^2^ in the hypothermia phase and 281 mL/min/m^2^ for rewarming. Moreover, an individualized critical DO_2_i threshold should be advocated during CPB.

## 1 Introduction

Acute kidney injury (AKI) is the most common complication in patients undergoing cardiac surgery with cardiopulmonary bypass (CPB), and is associated with increased mortality and morbidity (1, 2). Even mild AKI increases the risk of long-term adverse kidney events (3). Nadir indexed oxygen delivery (DO_2_i) during CPB is identified as a potentially modifiable risk factor for postoperative AKI (4). Previous observational studies have determined the critical threshold of DO_2_i to avoid postoperative AKI, in the range of 225–300 mL/min/m^2^ during CPB (5–8). Goal-directed perfusion (GDP) aimed to maintain DO_2_i above a critical threshold during CPB, reducing the incidence of AKI by approximately 50% (9). Moreover, the predictive capacity of DO_2_i on AKI was validated in an AKI prediction model (10), which was established from a large sample, with a DO_2_i threshold of 270 mL/min/m^2^.

About 30–60% of children develop AKI after congenital heart surgery, which is significantly associated with postoperative adverse outcomes (11, 12). Infants may be more susceptible to AKI after pediatric cardiac surgery, for both younger age and lower body weight are independent risk factors (11, 13). However, the critical DO_2_i for preventing AKI in children has not been well studied, and the published results remain controversial (14, 15). The objective of the present study was to explore the association between nadir DO_2_i and postoperative AKI in low-weight infants (≤10 kg) undergoing cardiac surgery with CPB.

## 2 Materials and methods

### 2.1 Study design and patients

This was a prospective observational study conducted at Fuwai hospital (Beijing, China). From August 2021 to July 2022, every infant scheduled for open-heart cardiac surgery was screened for eligibility. The criteria for selecting the participants were low-weight infants (≤10 kg) undergoing cardiac surgery with an expected CPB duration of 1–3 h (based on surgical complexity). Exclusion criteria were as follows: preoperative complications (chronic kidney disease or central nervous system injury), previous cardiac surgery, expected circulatory arrest during CPB, intraoperative ECMO, repeated CPB, aortic cross-clamping (ACC) duration <30 min or >120 min, and nadir rectal temperature <26°C during CPB.

The study has been approved by the Medical Ethics committee of Fuwai Hospital, Chinese Academy of Medical Sciences and Peking Union Medical College (Approval number: 2021-1483). Written informed consent was obtained from the legal guardian of every enrolled subject. This study followed STROBE reporting guidelines.

### 2.2 Data collection and definitions

The following variables were collected. Preoperative data: patient demographics and baseline laboratory tests, including Hb, serum creatinine (SCr), and blood urea nitrogen (BUN). Intraoperative data: type of surgery, European Association for Cardiothoracic Surgery-Society of Thoracic Surgeons (STS–EACTS) categories, CPB duration, ACC duration, volume of RBC consumption (including RBC added in CPB priming), and nadir DO_2_i in the hypothermia and rewarming phases of CPB. DO_2_i was calculated manually following the formula: DO_2_i (mL/min/m^2^) = indexed pump flow (L/min/m^2^) × [1.36 × Hb (g/L) × Hb saturation (%) + 0.031 × partial pressure of arterial oxygen (mmHg)]. The blood gas analysis was routinely monitored at the following time points: after cardioplegia, every 30 min during ACC, at rewarming, and aortic unclamping. Postoperative variables collected included: duration of mechanical ventilation (MV), length of stay (LOS) in PICU, hospital LOS, AKI, and in-hospital adverse events. In addition, after weaning from CPB, plasma-free hemoglobin (pFHb) was measured using the Plasma/Low Hb Photometer (HemoCue AB, Ängelholm, Sweden).

In view of the correlation between body temperature and oxygen metabolism, patients were divided into mild hypothermia (32–34°C) and moderate hypothermia (26–32°C) according to the lowest rectal temperature. We determined postoperative AKI by the Kidney Disease Improving Global Outcomes (KDIGO) criteria (16). Severe hemolysis after CPB was defined as pFHb > 500 mg/L (17).

### 2.3 Clinical practice

General anesthesia was performed under the standard protocol of the institution. The standard CPB circuit consists of a roller pump (Stockert S5, Sorin, Germany), a hollow fiber membrane oxygenator (Fx05, Terumo, Japan), and a hemofilter (Maquet BC20, Hirrlingen, Germany). The priming volume of the pump circuit was approximately 200 mL, including Acetated Ringer’s solution, packed red blood cells (RBC), Gelofusine, and sodium bicarbonate. According to the expected CPB duration, patients were treated with modified St Thomas solution or histidine-tryptophan-ketoglutarate solution for cold cardioplegia. The target hypothermia was determined by the surgical complexity and reflux from collateral arteries during CPB.

All patients received arterial pump flow based on body weight and temperature, with a target value of 100–150 mL/kg/min during the hypothermia phase, and 120–180 mL/kg/min during the rewarming phase. Mean arterial pressure was maintained at 30–50 mmHg, and the lowest hemoglobin (Hb) threshold was 70 g/L during CPB. Patients were rewarming to the nasopharyngeal temperature above 36°C with a satisfactory hemodynamic state before weaning from CPB. Modified ultrafiltration (MUF) was routinely carried out to reach Hb > 100 g/L. After the operation, all patients were transferred to the pediatric intensive care unit (PICU).

### 2.4 Statistical analysis

The normal distribution of variables was assessed visually through Q-Q diagram and histogram. Continuous normal distribution variables were presented as mean ± standard deviation, and median with interquartile ranges for non–normally distributed variables, and were compared using the *T*-test or Mann–Whitney U test. Categorical variables were expressed as frequencies and percentages, and the Chi-square test or Fisher’s exact test was used for comparisons.

In order to explore the association between nadir DO_2_i and AKI, the following analysis was performed in patients with mild hypothermia and moderate hypothermia during CPB, respectively. Receiver operating characteristic (ROC) curve analysis was used to determine the predictive performance of hypothermia and rewarming nadir DO_2_i for postoperative AKI, and the cutoff value, sensitivity, and specificity were reported. Univariable and multivariable logistic regression was used to determine the effect of hypothermia and rewarming nadir DO_2_i on postoperative AKI. According to the risk factors associated with AKI in pediatric cardiac surgery, the following adjustment factors were selected: age, weight, baseline SCr, STS–EACTS categories, CPB duration, and pFHb (13, 18).

All statistical analyses were performed using SPSS 25.0 and R 4.1.0, and a *p*-value < 0.05 was considered statistically significant.

## 3 Results

### 3.1 Study population

**FIGURE 1:**
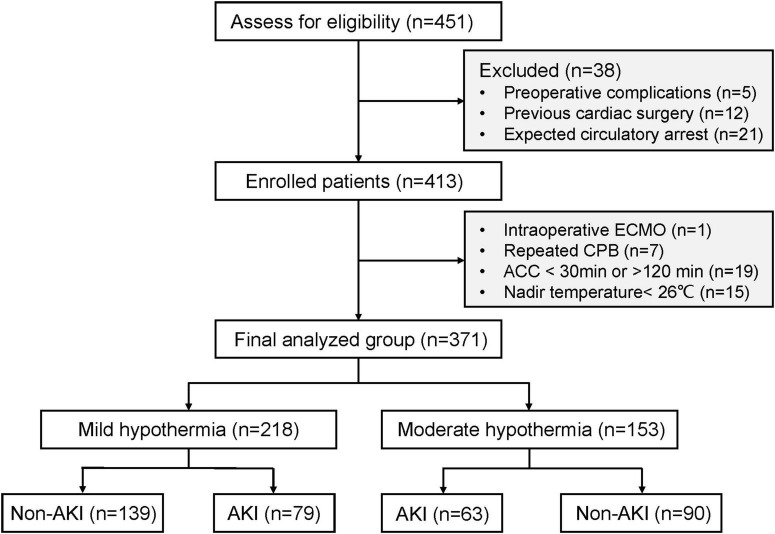
Flow chart of the study.

From August 2021 to July 2022, a total of 413 patients were enrolled. 42 patients were excluded, including intraoperative ECMO (*n* = 1), repeated CPB (*n* = 7), ACC duration <30 min or >120 min (*n* = 19), and nadir temperature < 26°C (*n* = 15). Finally, 371 patients were included in the analysis. Among them, 218 (58.8%) patients underwent mild hypothermia and 153 (41.2%) patients underwent moderate hypothermia during CPB (Figure 1).

Baseline characteristics of the patients included were demonstrated in Table 1. According to KDIGO criteria, 142 (38.3%) patients developed AKI after surgery.

**TABLE 1:** Baseline characteristics of the patients.

Variables	Non-AKI (*n* = 229)	AKI (*n* = 142)	*P*-value
Age (day)	197 (131–302)	180 (120–258)	0.071
Male	119 (52.0%)	72 (50.7%)	0.813
Weight (kg)	7.05 ± 1.62	6.74 ± 1.69	0.086
Body length (cm)	67.89 ± 7.28	66.07 ± 7.85	0.024
BSA (m^2^)	0.34 ± 0.6	0.32 ± 0.6	0.020
Gestation (week)	38.61 ± 1.86	38.24 ± 2.28	0.091
Birth weight (kg)	3.17 ± 0.57	3.09 ± 0.67	0.205
**perative laboratory test**			
Hemoglobin (g/L)	115.53 ± 16.46	111.35 ± 14.11	0.013
Albumin (g/L)	41.99 ± 3.24	42.18 ± 3.21	0.589
Serum creatinine (umol/L)	29.63 ± 8.40	23.57 ± 5.97	<0.001
Cystatin-C (mg/L)	1.12 ± 0.24	1.10 ± 0.21	0.312
Blood urea nitrogen (umol/L)	3.27 (2.27–4.64)	3.01 (2.23–4.5)	0.338
**Type of surgery**			
ASD/VSD	140 (61.1%)	87 (61.3%)	
TOF/Pulmonary stenosis	36 (15.7%)	22 (15.5%)	
Valvuloplasty (mitral/aortic)	24 (10.5%)	25 (17.6%)	
TECD	9 (3.9%)	4 (2.8%)	
TAPVC	9 (3.9%)	3 (2.1%)	
DORV	9 (3.9%)	0 (0%)	
Pulmonary atresia	2 (0.9%)	1 (0.7%)	
STS–EACTS categories	1.52 ± 0.76	1.42 ± 0.55	0.177

AKI, acute kidney injury; BSA, body surface area; ASD, atrial septal defect; VSD, ventricular septal defect; TOF, Tetralogy of Fallot; TECD, total endocardial cushion defect; TAPVC, total anomalous pulmonary venous connection; DORV, double outlet right ventricle; EACTS, European Association for Cardiothoracic Surgery; STS, Society of Thoracic Surgeons.

The differences between groups showed that AKI patients had lower BSA, body length, baseline SCr, and Hb. There was no significant difference in STS–EACTS categories of cardiac surgery. The types of surgery were also shown in Table 1.

### 3.2 Intraoperative data and clinical outcomes

Patients who developed AKI had lower nadir DO_2_i during the hypothermic phase (*p* = 0.034) and rewarming phase (*p* < 0.001) of CPB. The nadir temperature of AKI patients was lower albeit it was not statistically significant (30.98 ± 1.13 vs 31.20 ± 1.05, *p* = 0.064). There was no significant difference in other intraoperative data between the two groups, including duration of CPB and ACC, RBC consumption, and pFHb after CPB. Patients with AKI experienced prolonged MV (*p* = 0.014). There was no significant difference in PICU LOS, hospital LOS, and postoperative adverse events between patients with and without AKI (Table 2).

**TABLE 2:** Intraoperative data and clinical outcomes.

Variables	Non-AKI (*n* = 229)	AKI (*n* = 142)	*P*-value
**Intraoperative data**			
CPB duration (min)	87.49 ± 30.43	85.82 ± 28.70	0.60
ACC duration (min)	60.19 ± 22.94	59.15 ± 22.48	0.669
RBC consumption (U)	1.01 ± 0.47	1.06 ± 0.52	0.361
Nadir temperature on CPB (°C)	30.98 ± 1.13	31.20 ± 1.05	0.064
Hypothermic nadir DO_2_i (mL/min/m^2^)	256.15 ± 41.48	247.18 ± 35.84	0.034
Rewarming nadir DO_2_i (mL/min/m^2^)	286.85 ± 40.63	269.77 ± 37.35	<0.001
pFHb after CPB (mg/L)	400 (200–400)	400 (200–400)	0.964
Conventional ultrafiltration (mL)	229.48 ± 108.0	240.56 ± 107.17	0.336
Modified ultrafiltration (mL)	124.54 ± 56.20	125.28 ± 54.24	0.901
**Postoperative outcomes**			
Mechanical ventilation (h)	10 (6–24)	19 (7–26)	0.014
Length of PICU stay (d)	3 (2–5)	3 (2–5)	0.838
Length of hospital stay (d)	13 (10–17)	13 (9.75–16)	0.579
Postoperative RBC transfusion (U)	0.52 ± 0.61	0.60 ± 0.74	0.276
Adverse events (%)	15 (6.6%)	12 (8.5%)	0.493
Peritoneal dialysis	3 (1.3%)	3 (2.1%)	0.551
Respiratory infection	4 (1.7%)	5 (3.5%)	0.289
Re-intubation	5 (2.2%)	2 (1.4%)	0.586
Re-operation	2 (0.9%)	4 (2.8%)	0.156
ECMO	1 (0.4%)	1 (0.7%)	0.736
In-hospital mortality	0 (0%)	1 (0.7%)	0.165

AKI, acute kidney injury; CPB, cardiopulmonary bypass; ACC, aortic cross-clamping; RBC, red blood cell; DO_2_i, indexed oxygen delivery; pFHb, plasma free hemoglobin; PICU, pediatric intensive care unit; ECMO, extracorporeal membrane oxygenation.

### 3.3 Nadir DO_2_i in hypothermic and rewarming phase

**FIGURE 2:**
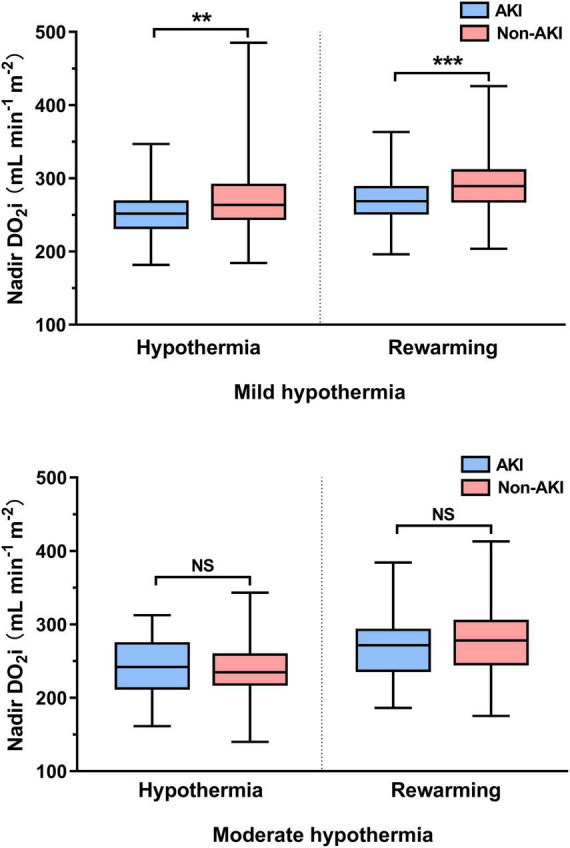
Hypothermic and rewarming nadir indexed oxygen delivery (DO_2_i) in mild and moderate hypothermia. NS, not significant (*p* > 0.05); **p < 0.01; ***p < 0.001.

According to the lowest rectal temperature, patients were divided into mild hypothermia (32–34°C) and moderate hypothermia (26–32°C). In patients undergoing mild hypothermia, nadir DO_2_i was significantly decreased, whether in the hypothermia or rewarming phase of CPB. However, these differences were not significant in patients with moderate hypothermia during CPB (Figure 2).

**FIGURE 3:**
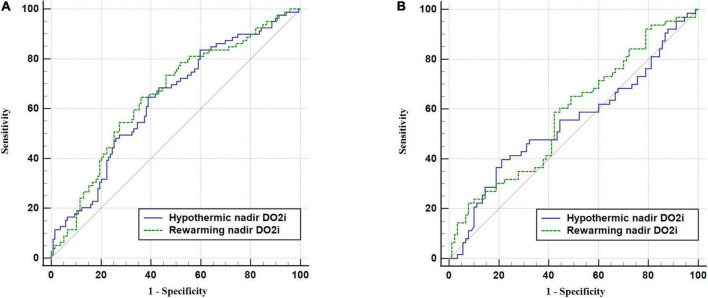
Receiver operating characteristic (ROC) curve of nadir DO_2_i in hypothermic and rewarming phases for postoperative AKI. **(A)** Patients undergoing mild hypothermia during CPB. **(B)** Patients undergoing moderate hypothermia during CPB.

To assess the predictive performance of nadir DO_2_i for AKI, ROC curve analysis was conducted in patients undergoing mild hypothermia (Figure 3A) and moderate hypothermia (Figure 3B) during CPB, respectively. In patients undergoing mild hypothermia during CPB, hypothermia nadir DO_2_i had an AUC of 0.64 (95% CI 0.57–0.70, *p* < 0.001), with a cutoff value of 258 mL/min/m^2^ (sensitivity 64.6% and specificity 61.2%). The better predictor was rewarming nadir DO_2_i, which had an AUC of 0.65 (95% CI 0.59–0.72, *p* < 0.001), with a cut-off value of 281 mL/min/m^2^ (sensitivity 64.6% and specificity 64.0%). In patients undergoing moderate hypothermia during CPB, neither hypothermia nadir DO_2_i (*p* = 0.348) nor rewarming nadir DO_2_i (*p* = 0.086) showed predictive performance for postoperative AKI (Table 3).

**TABLE 3:** Receiver operating characteristic (ROC) curve of nadir DO_2_i in hypothermic and rewarming phases for postoperative AKI.

	Cut-off value	AUC (95%CI)	*P*-value
**Mild hypothermia**			
Hypothermic phase	258.05	0.637 (0.569–0.701)	<0.001
Rewarming phase	280.98	0.653 (0.585–0.716)	<0.001
**Moderate hypothermia**			
Hypothermic phase	–	0.546 (0.464–0.627)	0.348
Rewarming phase	–	0.581 (0.498–0.660)	0.086

ROC, receiver operating characteristic; DO_2_i, indexed oxygen delivery; AKI, acute kidney injury; AUC, area under the curve.

**FIGURE 4:**
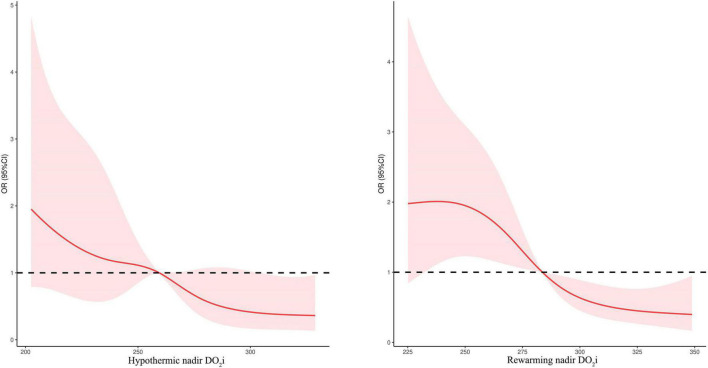
Relationship between postoperative AKI and nadir DO_2_i during the hypothermic and rewarming phases.

Considering the correlation between nadir DO_2_i and postoperative AKI in patients undergoing mild hypothermia during CPB, we further evaluated the association using univariable and multivariable logistic regression. The relationship between postoperative AKI and nadir DO_2_i during the hypothermia and rewarming phases was shown in Figure 4.

As shown in Table 4, after adjusting age, weight, baseline SCr, STS–EACTS categories, CPB duration, and pFHb in multivariable analysis, nadir DO_2_i during hypothermia and rewarming phases was associated with postoperative AKI, respectively (*p* = 0.001). When both the hypothermia and rewarming nadir DO_2_i were brought into logistic regression using a backward stepwise variable selection, the latter remained in the final model. Rewarming nadir DO_2_i also demonstrated a higher prediction probability in univariable analysis (ROC-AUC: 0.653 vs 0.637). When nadir DO_2_i was converted into categorical variables according to the cutoff values, hypothermia nadir DO_2_i < 258 mL/min/m^2^ (OR 3.012, 95%CI 1.635–5.548, *p* < 0.001) and rewarming nadir DO_2_i < 281 mL/min/m^2^ (OR 3.246, 95%CI 1.767–5.963, *p* < 0.001) were independent risk factors for postoperative AKI (Table 4). Moreover, in patients undergoing moderate hypothermia, hypothermia nadir DO_2_i (*p* = 0.324) and rewarming nadir DO_2_i (*p* = 0.069) were not associated with postoperative AKI.

**TABLE 4:** Univariable and multivariable analysis of nadir DO_2_i and AKI in patients undergoing mild hypothermia.

	OR	95% CI	*P*-value
**Hypothermic nadir DO_2_i (mL/min/m^2^)**			
Unadjusted	0.986	0.978–0.994	0.001
Multivariable adjusted	0.986	0.977–0.994	0.001
**Hypothermic nadir DO_2_i < 258 mL/min/m^2^**			
Unadjusted	2.714	1.534–4.802	0.001
Multivariable adjusted	3.012	1.635–5.548	<0.001
**Rewarming nadir DO_2_i (mL/min/m^2^)**			
Unadjusted	0.985	0.977–0.993	<0.001
Multivariable adjusted	0.986	0.977–0.995	0.001
**Rewarming nadir DO_2_i < 281 mL/min/m^2^**			
Unadjusted	3.242	1.821–5.771	<.001
Multivariable adjusted	3.246	1.767–5.963	<0.001

DO_2_i, indexed oxygen delivery; AKI, acute kidney injury; OR, odds ratio; CI, confidence interval.

### 3.4 Correlation between nadir DO_2_i and pFHb after CPB

After weaning from CPB, 108 (29.1%) patients developed severe hemolysis (pFHb > 500 mg/L). Hypothermia and rewarming nadir DO_2_i were comparable between patients with or without severe hemolysis (Supplementary Table 1). There was no correlation between pFHb and nadir DO_2_i in patients undergoing mild or moderate hypothermia during CPB (Supplementary Table 2).

## 4 Discussion

In this study, we demonstrated that nadir DO_2_i during CPB was associated with postoperative AKI in low-weight infants undergoing mild hypothermia cardiac surgery. The critical DO_2_i for preventing AKI was 258 mL/min/m^2^ during the hypothermia phase and 281 mL/min/m^2^ for rewarming. In contrast, rewarming nadir DO_2_i was a slightly better predictor of postoperative AKI. In addition, there was no significant association between nadir DO_2_i and postoperative AKI in patients undergoing moderate hypothermia during CPB.

The association between DO_2_i during CPB and AKI is one of the hot topics in cardiac surgery. Previous retrospective studies determined that the critical DO_2_i for avoiding AKI was approximately 270 mL/min/m^2^ in adults (6, 7, 10). Subsequently, GDP management aimed at maintaining the DO_2_i level above the specific critical threshold was proposed (19). A meta-analysis of existing research suggested that GDP based on active DO_2_i management effectively reduced the incidence of AKI after cardiac surgery (9). As a simple and effective management strategy, the application of GDP during CPB was recommended in the recent clinical practice guidelines (4).

Compared with adults, pediatric cardiac surgery is highly heterogeneous in terms of surgical complexity and physiological characteristics. A general threshold of DO_2_i might not apply to different types of children, such as newborns, infants and preschoolers (15). Bojan and his colleagues indicated that 340 mL/min/m^2^ was the lowest suitable DO_2_i for neonates to maintain aerobic metabolism during normothermic CPB (20). However, the primary endpoint of this study was hyperlactatemia after aortic clamping, which was of limited value in preventing postoperative AKI. Zhang et al. (14) reported that the threshold of DO_2_i of children should be higher than that in adults because of the higher metabolic rate. They recommended a critical value of 353 mL/min/m^2^ to prevent postoperative AKI. However, the threshold of 353 mL/min/m^2^ was significantly higher than that of our clinical practice. Therefore, we published correspondence (15) and conducted this prospective observational study. The enrolled subjects were low-weight infants like their study, because these patients have the same CPB circuit composition and management strategy, and are more susceptible to postoperative AKI (11, 13).

The most likely explanation for the disparity in nadir DO_2_i was the differences in CPB management strategy, which were mainly reflected in the two important parameters in the calculation formula of DO_2_i: Hb and pump flow rate. In recent years, our institution has adopted restrictive patient blood management to minimize allogeneic RBC transfusion. Previous literature has proved the safety of the conservative transfusion strategy with a Hb threshold of 70 g/L during CPB (21). Additionally, we adopted a miniaturized CPB circuit to matched the blood conservation strategy and correspondingly reduced pump flow rates. Intraoperative oxygen delivery-consumption balance was ensured by monitoring mean arterial pressure, mixed venous oxygen saturation, regional cerebral oxygen saturation, lactate, and urine output. A recent study also indicated that while maintaining normal hemodynamic and metabolic parameters, reducing pump flow would not damage renal function and in-hospital outcomes (22). As a cardiac center that performs more than 4,000 pediatric cardiac surgery every year, our current standard CPB protocol has been tested through years of clinical practice. The incidence of postoperative AKI in our patients (38.3%) was comparable to previous reports (14, 23), even though our subjects were low-weight infants.

Different from previous studies (14, 20), we analyzed the nadir DO_2_i during hypothermia and rewarming phases, respectively. This allowed better differentiation of the nadir DO_2_i during the whole CPB period, and thus better matched the actual dynamic flow management. The importance of rewarming DO_2_i should be emphasized because the rewarming phase had greater risk of medullary hypoxia and AKI (24, 25). Even with high oxygen delivery during hypothermia, microcirculatory perfusion disturbances-induced impaired oxygen consumption would cause oxygen debt (26, 27). In contrast, during the rewarming phase, increasing oxygen delivery levels might recruit and perfuse more vascular beds (28, 29).

In our CPB circuit, it is difficult to provide the same flow rate as that in Zhang et al.’s research (2.8 ∼ 3.2 L/min/m^2^) (14) at a safe fluid level in the oxygenator and appropriate pump pressure. Because of the concern that high DO_2_i might lead to increased blood destruction, we measured pFHb after weaning from CPB to partially represent the degree of blood destruction. However, there was no correlation between nadir DO_2_i and pFHb. This might suggest that there would be no difference in blood destruction under CPB management strategies based on different critical DO_2_i values. Generally, CPB circuits were similar in adults, while pediatric CPB circuits had various components and corresponding management strategies in different institutions, including the management of hematocrit, body temperature and pump flow rate. Applying a general pediatric DO_2_i threshold such as 350 mL/min/m^2^ across different institutions is unrealistic in many cases. Therefore, pediatric GDP based on DO_2_i might not have to follow a fixed protocol. Under the strategy of evidence-based CPB management, pediatric GDP based on actual clinical practice would benefit the patient.

There were some limitations. Firstly, our study was a prospective observational design with no intervention. Further well-designed randomized controlled trials are needed to determine the effect of DO_2_i-based GDP on preventing postoperative AKI in pediatric cardiac surgery. Additionally, as DO_2_i was not continuously monitored, the nadir DO_2_i analyzed might not be the actual lowest value. Nevertheless, our results are still helpful for most institutions where continuous DO_2_i monitoring is not available.

## 5 Conclusion

In low-weight infants undergoing mild hypothermia during CPB, nadir DO_2_i was associated with postoperative AKI. The critical DO_2_i for preventing AKI was 258 mL/min/m^2^ during the hypothermia phase and 281 mL/min/m^2^ for rewarming. It should be highlighted that higher oxygen delivery might be required during rewarming phase to avoid hypoxia and hypoperfusion. The critical DO_2_i threshold during CPB to prevent postoperative AKI should be individualized according to institutional clinical practice.

## Data availability statement

The raw data supporting the conclusions of this article will be made available by the authors, without undue reservation.

## Ethics statement

The studies involving human participants were reviewed and approved by the Medical Ethics Committee of Fuwai Hospital. Written informed consent to participate in this study was provided by the participants’ legal guardian/next of kin.

## Author contributions

PG, YJ, PZ, WW, JH, and JL contributed to the conception and design of the study. PG, JH, and YJ organized the database. PG and PZ performed the statistical analysis. PG wrote the first draft of the manuscript. JH and JL revised the manuscript. All authors contributed to the manuscript revision, read, and approved the submitted version.
