# Supplementary material for: Nadir oxygen delivery is associated with postoperative acute kidney injury in low-weight infants undergoing cardiopulmonary bypass
Source: Front Cardiovasc Med. 2022 Dec 16;9:1020846. doi: 10.3389/fcvm.2022.1020846 (PMC9800598; doi:10.3389/fcvm.2022.1020846)
Supplement: Supplementary file 1 [file Table_1.DOCX]

**Supplementary Table 1.** Comparison of nadir DO_2_i between patients with severe hemolysis

| Nadir DO_2_i (mL/min/m^2^) | Severe hemolysis  (n = 108) | Non-Severe hemolysis  (n = 263) | *P* value |
| --- | --- | --- | --- |
| **Mild hypothermia** | | | |
| Hypothermic | 254.61 ± 31.17 | 263.90 ± 40.53 | 0.140 |
| rewarming | 282.67 ± 40.53 | 285.71 ± 38.50 | 0.622 |
| **Moderate hypothermia** | | | |
| Hypothermic | 237.69 ± 41.57 | 241.06 ± 34.43 | 0.588 |
| rewarming | 279.81 ± 42.47 | 269.67 ± 42.37 | 0.152 |

DO_2_i, indexed oxygen delivery.

**Supplementary Table 2.** Correlation between nadir DO_2_i and pFHb after CPB

| Nadir DO_2_i (mL/min/m^2^) | Correlation coefficient | *P* value | |
| --- | --- | --- | --- |
| **Mild hypothermia** | | |  |
| Hypothermic | -0.103 | 0.130 | |
| rewarming | -0.025 | 0.710 | |
| **Moderate hypothermia** | | | |
| Hypothermic | -0.063 | 0.440 | |
| rewarming | 0.116 | 0.155 | |

DO_2_i, indexed oxygen delivery, pFHb, plasma free hemoglobin.
